# Amantadine against glioma via ROS-mediated apoptosis and autophagy arrest

**DOI:** 10.1038/s41419-024-07228-x

**Published:** 2024-11-15

**Authors:** Yusong Luo, Ruolan Liu, He Zhang, Hongyu Wang, Hang Yin, Guopeng Tian, Bo Wang, Yunji Yan, Zilin Ding, Junqiang Dai, Liang Niu, Guoqiang Yuan, Yawen Pan

**Affiliations:** 1 Department of Neurosurgery, the Second Hospital & Clinical Medical School, Lanzhou University, Lanzhou, China; 2 Gansu Provincial Clinical Research Center for Neurological Diseases, the Second Hospital & Clinical Medical School, Lanzhou University, Lanzhou, China; 3 Academician Workstation, the Second Hospital & Clinical Medical School, Lanzhou University, Lanzhou, China

**Keywords:** Drug development, CNS cancer

## Abstract

Glioma is a common primary nervous system malignant tumor with poor overall cure rate and low survival rate, yet successful treatment still remains a challenge. Here, we demonstrated that amantadine (AMT) exhibits the powerful anti-glioma effect by promoting apoptosis and autophagy in vivo and in vitro. Mechanistically, amantadine induces a large amount of reactive oxygen species (ROS) accumulation in glioma cells, and then triggers apoptosis by destroying mitochondria. In addition, amantadine induces the initiation of autophagy and inhibits the fusion of autophagosome and lysosome, consequently performing an anti-glioma role. Taken together, our findings suggest that amantadine could be a promising anti-glioma drug that inhibits glioma cells by inducing apoptosis and autophagy, which may provide a novel potential treatment option for patients.

## Introduction

Glioma as a common primary and aggressive brain tumor accounts for about 70% of primary malignant brain tumors corresponding to the American Brain Tumor Registry [1, 2]. In the past 30 years, the incidence of glioma has been rising year by year, and its treatment is still dominated by surgery, radiotherapy and chemotherapy. Glioma has no obvious boundary with normal brain tissue, so that surgery is hard to completely remove it, and radiation therapy has not significantly enhanced the treatment effect [3]. At present, the first-line treatment drug temozolomide (TMZ) can effectively improve glioma and astrocytoma with low expression of DNA repair enzyme O6-methylguanine-DNA-methyltransferase (MGMT), while the clinical treatment for glioma with high MGMT expression is still lacking [4]. Glioma is highly heterogeneous, and chemotherapy based on a single mechanism can easily lead to drug resistance and relapse. Therefore, new therapeutic agents are required to improve the clinical outcome of glioma patients [5].

Apoptosis plays an important role in maintaining homeostasis and normal operation of the body, and the whole process is complex and orderly. Apoptosis as one of the ways of programmed active cell death triggers the activation of a series of signal pathways and induces cell morphological changes mainly by controlling gene expression, thereby promoting the spontaneous and orderly death of cell [6]. Apoptosis is triggered mainly by an exogenous pathway or an endogenous pathway, and the two pathways converge to activate Caspase to cause morphological and molecular biological changes of the cell so that the cell eventually dies [7]. The exogenous pathway triggers apoptosis through the ligand binding to the death receptor in cell membrane [8]. The endogenous pathway is generally induced by DNA damage, chemoradiotherapy, overheating, viral infection and other factors, which leads to the damage of mitochondria or the change of mitochondria membrane permeability in cells, thus inducing the occurrence of apoptosis [9]. Oxidative stress is often associated with the development of tumor cells. When a large amount of intracellular ROS accumulation leads to oxidative stress, it can trigger the occurrence of endogenous apoptosis via destroying mitochondrial. Therefore, elucidation of the molecular mechanism of apoptosis in anti-tumor drugs can offer a new scheme to the treatment of tumor.

Autophagy is the process of degradation and reuse of aging or damaged organelles and biomacromolecules within cells. The process of autophagy can be divided into several key stages, including the formation of Autophagosome and the inclusion of contents, the fusion of autophagosome and lysosome to form Autolysosome, and the degradation of contents [10, 11]. Many anti-tumor drugs can promote autophagy, and autophagy shows different effects in tumor chemotherapy. On the one hand, some drugs can induce protective autophagy by clearing the damaged biomacromolecules produced during radiotherapy and chemotherapy, promoting the survival of tumor cells and reducing the anti-tumor effect of drugs [12, 13]. On the other hand, several drugs can induce autophagy cell death, consequently enhancing the killing effect of drugs on tumor cells [14, 15]. Therefore, exploring the molecular mechanism and biological function of autophagy in anti-tumor drugs is helpful to provide a new strategy for improving the anti-tumor effect of drug combination from the perspective of inhibiting protective autophagy or enhancing autophagy death.

Amantadine, the first FDA-approved antiviral drug for the treatment of influenza A virus infection, inhibits the replication of influenza A virus RNA by preventing the dissociation of M1 protein from ribonucleoside protein [16, 17]. Amantadine as an N-methyl-D-aspartic acid receptor (NMDAR) antagonist is also used to treat intractable dyskinesia and palsy tremor by promoting the synthesis of peripheral nerve catecholamine and inhibiting its reuptake, and by reactivating cholinergic neurons [18, 19]. In recent years, it has been found that amantadine has a good anti-tumor effect in different tumor cells. For instance, amantadine has an anti-proliferation effect in melanoma cells, which promotes the apoptosis of tumor cells by increasing the Bax/Bcl-2 ratio and stops the cell cycle in G1/S phase [20].

Although amantadine can inhibit liver cancer cells by blocking cell cycle and inducing cell apoptosis [21], its application in glioma has not been reported so far. In this study, we sought to repurpose amantadine to explore the efficacy in the treatment of glioma and investigate the molecular mechanisms to find a new therapeutic strategy for glioma.

## Materials and methods

### Cell culture

U87 and U251 glioma cell lines were procured from the Chinese Academy of Sciences. Cells were incubated in DMEM (Gibco) containing 1% penicillin−streptomycin and 10% fetal bovine serum (FBS, BI) in a humidified incubator at 37 °C with 5% CO_2_.

### Reagents and antibodies

AMT (A136871) was purchased from Shanghai Aladdin Biotech Co., Ltd. 3-methyladenine (HY-19312), Ferrostatin-1 (HY-100579) and Z-VAD (HY-16658B), were obtained from Med Chem Express. Dimethylsulfoxide (DMSO), N-acetyl cysteine (NAC), and chloroquine (C6628) were bought from Millipore Sigma. Annexin V-FITC/PI Apoptosis Detection Kit (Catalog no. 40302ES60) was purchased from Yeasen Biotech Co., Ltd. ROS assay kit (Catalog no. s0033s), JC-1 (Catalog no. C2006), LDH (Catalog no. C0016) were provided by Beyotime Biotechnology. The following antibodies were used: anti-β-actin (T40104) was purchased from Abmart; Cleaved-PARP (9532), ATG5 (12994S), ATG7 (8558S), P62 (23214S), Cleaved-caspase3 (9661S) and LC3 (3868S) were obtained from Cell Signaling Technology; anti-Ki-67 (bsm-33070 M) was purchased from Bioss.

### Cell viability

U87 and U251 cells were seeded in 96-well plate (1 × 10^4^ cells/well) and incubated for 12 h, respectively. Then, the culture solution was changed to fresh medium containing different concentrations of AMT (0, 20, 40, 60, 80 μM) for 24 h. After that, the cell viability rate was evaluated using a standard CCK-8 assay. The cell viability (%) was calculated by average OD sample/average OD control × 100%.

### LDH release assay

U87 and U251 cells were seeded in 96-well plates (4000 cells/well) and incubated to about 80% confluency. Then, the cells were treated with indicated concentrations of AMT. LDH release ratios were measured using an LDH test kit (Beyotime, C0017).

### EdU Assay

U87 and U251 cells were seeded in 96-well plates and treated with the indicated concentrations of AMT for 24 h. The ratio of actively proliferating cells was conducted by the EdU Cell Proliferation Kit (Sangon, E607204).

### Colony Formation Assay

The long-term effects on antitumor ability were assessed by a colony formation assay. In brief, U87 and U251 cells were seeded in 24-well plates (500 cells/well). After 24 h, U87 and U251 cells were treated with the indicated concentration of AMT. After 2 weeks, the cell colonies were stained with crystal violet for 1 h and washed with PBS.

### Detection and analysis of apoptosis

Annexin V-FITC/PI Detection Kit was used to examine apoptotic cells following the manufacturer’s protocol. U87 and U251 cells (5 × 10^4^ cells per well) were seeded into 6-well plates and cultured overnight, then were subjected to the indicated concentrations of AMT. The treated cells were collected and washed with PBS. Then the cells were resuspended in binding buffer. Annexin V-FITC (5 μL) and PI (10 μL) were added into each tube. After incubation at room temperature for 15 min, apoptosis was detected by flow cytometry. All data from flow cytometry were analyzed using FlowJo software.

### Intracellular ROS generation assay

The DCFH-DA kit was utilized to measure the generation of intracellular ROS. U87 and U251 cells were cultured in 6-well plates (2 × 10^5^ cells/well) overnight, followed by treatment with varying concentrations of AMT. Subsequently, the cells were incubated with 1.0 μM DCFH-DA at 37 °C for 20 min. After two washes with PBS, both qualitative and quantitative assessments of intracellular ROS production were conducted using an inverted fluorescence microscope and flow cytometry, respectively.

### Mitochondrial membrane potential evaluation

To evaluate mitochondrial membrane potential, U87 and U521 cells were seeded into 6-well plates at a density of 5 × 10^4^ cells per well and cultured overnight. Then cells were subjected to the indicated concentrations of AMT. JC-1 solution was applied to stain the mitochondrial membrane for 20 min. The mitochondrial membrane potential was evaluated by fluorescence microscopy and flow cytometry according to the manufacturer’s protocol.

### Immunoblotting

Upon treatment, U87 and U251 cells were harvested and lysed in RIPA buffer (1% protease inhibitor cocktail) with sonication. And then, the samples were centrifuged at 10,000 rpm at 4 °C and boiled for 10 min. The samples were further analyzed through immunoblotting with specific antibodies.

### Immunofluorescence

U87 and U251 cells were incubated on sterilized cover slips in 24-well plates for 24 h, respectively. After indicated treatment, cells were fixed with 4% paraformaldehyde for 30 min, washed three times with PBS and permeabilized with 0.4% Triton X-100 and 5% BSA for 1.5 h, the slides were stained with primary antibodies at 4 °C overnight and subsequently incubated with secondary antibodies for 2 h at room temperature. After washing three times with PBS, nuclei were finally stained with DAPI for 8 min at room temperature. Images were visualized via by fluorescence microscopy.

### Animals studies

BALB/c nude mice (5 weeks, 18–20 g each) were purchased from Yaokang Biotechnology Co, Ltd. All animal studies were maintained in accordance with the National Institutes of Health Guide for the Care and Use of Laboratory Animals. The use of experimental animals was reviewed and approved by the Institutional Ethics Committee of Lanzhou University Second Hospital.

### In vivo Antitumor Study

For the orthotopic brain tumor model, 1 × 10^6^ U251 cells were intracranially engrafted in brains of BALB/c nude mice. Subcutaneous tumors were obtained by subcutaneous injection of U251 cells (5.0 × 10^7^ cells) into the right axilla in each BALB/c nude mouse. When the tumor size of the mice grew to approximately 80–100 mm^3^, the bearing mice were randomly divided into the following two groups (*n* = 5/group) (1): control (2), AMT treatment. Two groups were intraperitoneally injected with 100 μl of vehicle (10% ricinus oil, 5% DMSO, 10% ethanol, 75% physiologic saline) or AMT (50 mg/kg/day), respectively. The body weight and tumor volume of the treated mice were recorded every other day. The tumor volumes were calculated by the following formula: tumor volume (mm^3^) = (width^2^ × length)/2. The mice were euthanized at the end of treatment, following which the tumors were excised, weighed, photographed, and promptly fixed for subsequent immunohistochemical analysis. The various organs (heart, liver, spleen, lung and kidney) were collected and fixed accordingly. Subsequently, Hematoxylin-eosin (H&E) staining and Nissl staining were performed on these samples followed by microscopic imaging. The blood of mice was collected for measuring renal functions (CREA and UREA) and the serum levels of liver enzymes (ALT and AST) with an Assay Kit by an automatic biochemical analyzer.

### MRI (magnetic resonance imaging)

All MRI experiments were performed in a horizontal 30 cm bore 9.4 T system (uMR 9.4 T, United Imaging Life Science Instrument, Wuhan, China). Mice were anesthetized after 14-daytreatment and placed on the fixation system for imaging. The following sequence parameters were used: TR (repetition time) = 3000 ms; TE (echo time) = 49.28 ms; FA (flip angel) = 180°; matrix size = 208 × 208; FOV (field of view) = 20 × 20 mm.

### Statistical analysis

Quantitative data were expressed as mean ± SD. Statistical differences were made by one-way ANOVA and student’s t-test when comparing multiple or two groups, respectively. Statistical differences are denoted as. *P < 0.05, **P < 0.01, ***P < 0.001, (no significant, *p* > 0.05).

## Results

### AMT inhibits the growth of glioma cells in vitro

**Fig. 1:**
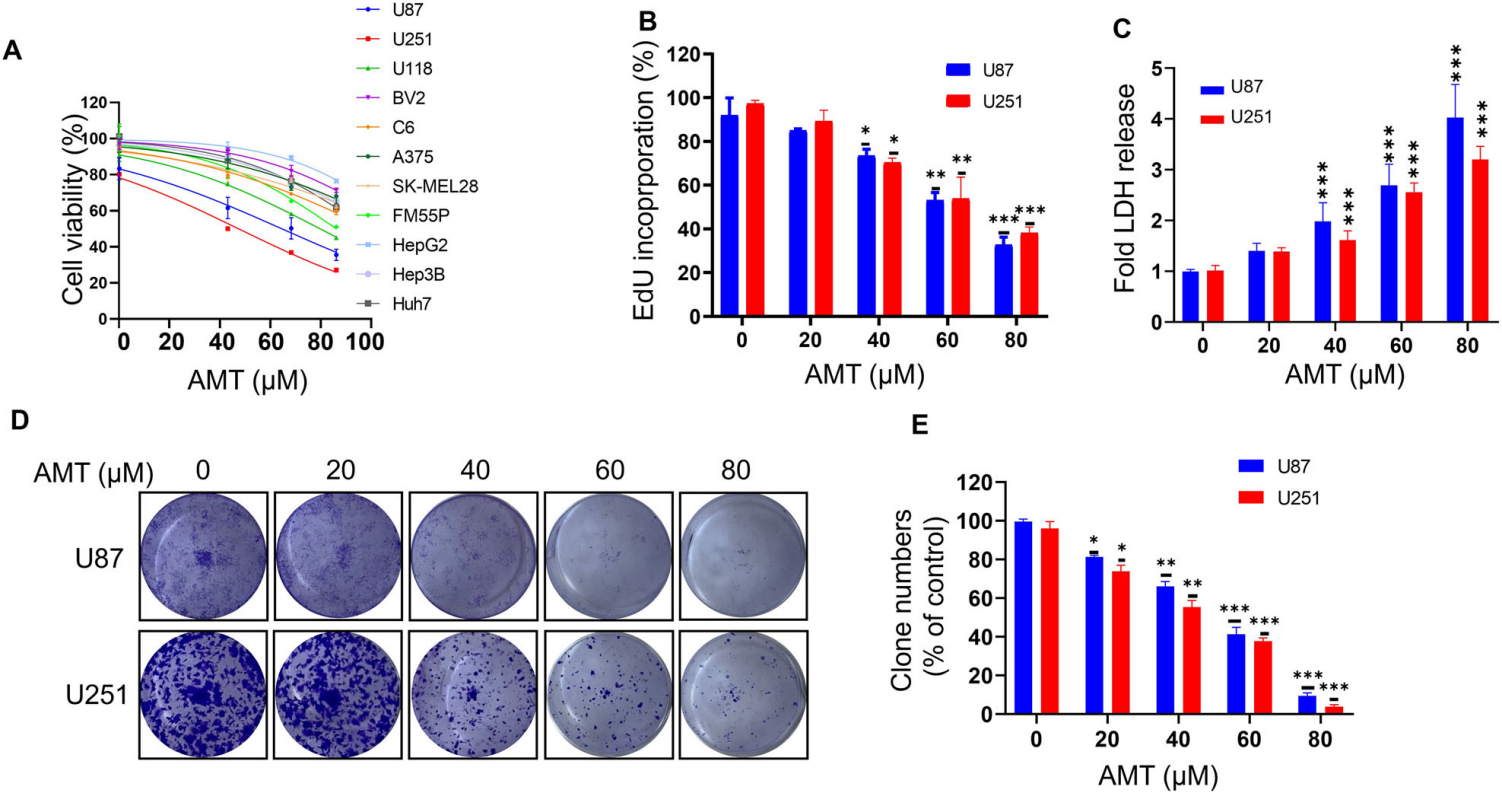
AMT repressed the cell viability of glioma cells. **A** The CCK-8 assay was performed in glioma cells (U87, U251, U118 and C6), mouse microglia (BV2), melanoma cells (A375, SK-MEL28 and FM55P) and hepatocellular carcinoma cells (HepG2, Hep3B and Huh7) treated with indicated concentrations of AMT for 24 h (*n* = 3). **B** Analysis of EdU incorporation in U87 and U251 treated with the indicated concentrations of AMT for 24 h (*n* = 3). **C** Analysis of LDH release in supernatants of U87 and U251 cells treated as in (**A**) for 24 h (*n* = 3). **D**, **E** Representative images for colony formation and quantitative analysis of U87 and U251 cells treated as in (**A**) for 24 h (*n* = 3). Error bars indicate mean ± SD (*P < 0.05, **P < 0.01, ***P < 0.001).

To validate the antitumor activity of AMT against tumor cell lines, we first detected cell growth after treatment with different concentrations of AMT. As depicted in Fig. 1A, we found that the IC50 of AMT in glioma tumors was lower than that in melanoma cancer and liver cancer, indicating that glioma cells may be more sensitive to AMT. Specifically, AMT treatment for 24 h markedly decreased the growth of various glioma cell lines, especially in U87 and U251. On the contrary, the immortalized BV2 microglial cells showed higher tolerance to AMT treatment. Consistently, the proliferation of glioma cells was also significantly decreased as evidenced by EdU incorporation (Figs. 1B and S1A). Moreover, the LDH release assay was performed to detect the cytotoxicity of AMT (Fig. 1C). As expected, AMT treatment markedly promoted the release of LDH in U87 and U251 cells. In addition, colony formation assay also indicated the proliferation-suppressive effect of AMT (Fig. 1D, E). Together, these data indicated that AMT significantly inhibits glioma cell growth in vitro.

### AMT suppresses glioma cell growth by triggering ROS-related apoptosis

**Fig. 2:**
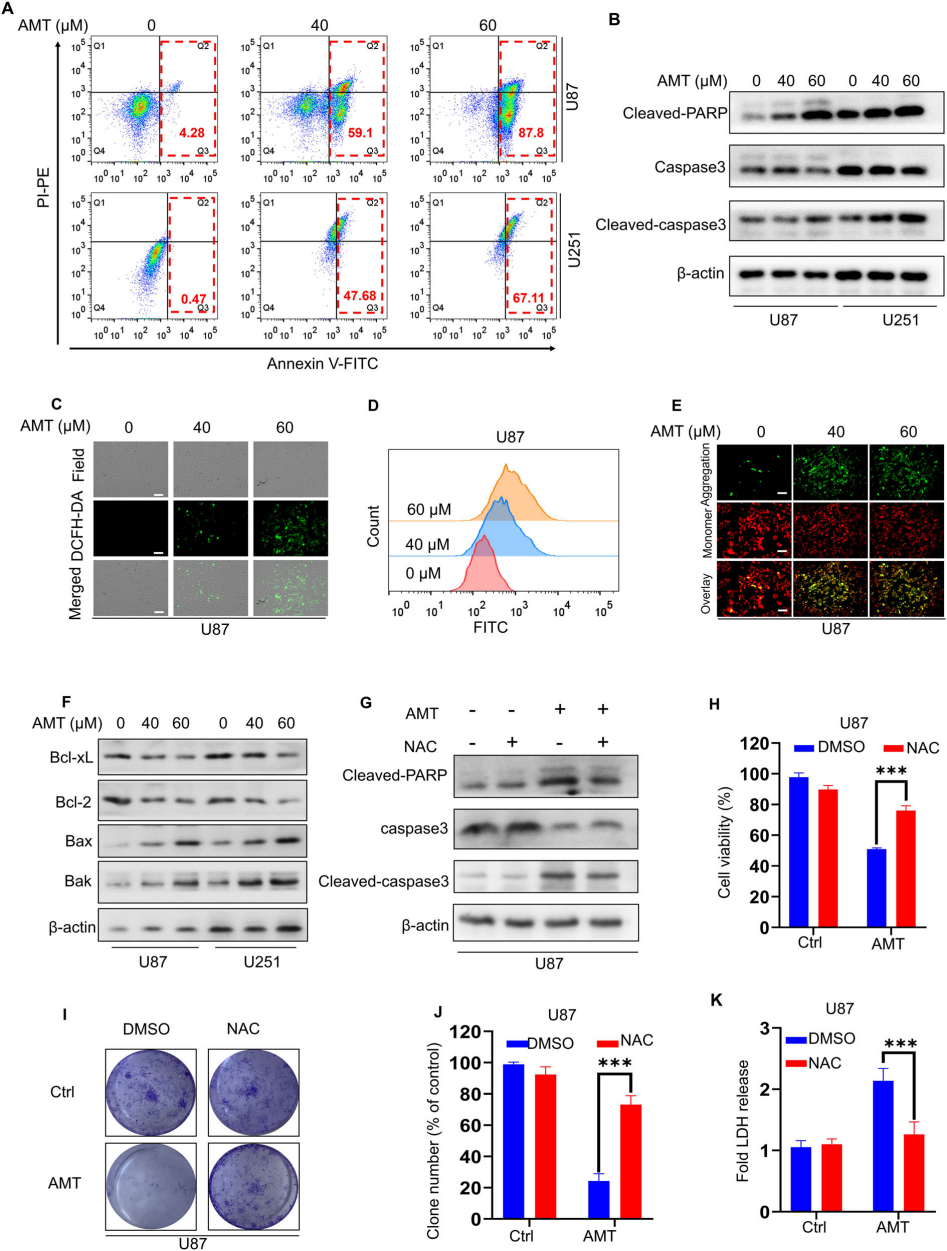
AMT induced excessive ROS accumulation is required for its apoptosis. **A** Annexin V-FITC/PI staining analysis of apoptosis U87 and U251 cells incubated with AMT at 0, 40, and 60 μM for 24 h by flow cytometry. **B** Immunoblotting analysis of cleaved-PARP, caspase3 and cleaved caspase3 in U87 and U251 cells treated as in (**A**) for 24 h. Fluorescence imaging (**C**) and flow cytometry analysis (**D**) for intracellular ROS level of U87 cells using DCFH-DA as a probe. Scale bar: 100 μm. **E** Fluorescence imaging for mitochondrial membrane potential of U87 cells. Scale bar: 100 μm. **F** Immunoblotting analysis of Bcl-xL, Bcl-2, Bax and Bak in U87 and U251 cells treated as in (**A**) for 24 h. **G** Immunoblotting analysis of cleaved-PARP, caspase3 and cleaved caspase3 in U87, cells were treated with 40 μM AMT in the presence or absence of NAC (2 mM). **H** Cell proliferation was examined by CCK-8 assay, cells were treated with 40 μM AMT in the presence or absence of NAC (2 mM) (*n* = 3). **I**, **J** Colony formation assay of U87 cells were treated as in (**G**) for 24 h (*n* = 3). **K** LDH release assay of U87 cells was treated as in (**G**) for 24 h (*n* = 3). Error bars indicate mean ± SD (***P < 0.001).

To elucidate the molecular mechanism underlying AMT-induced growth inhibition in glioma cells, we employed a combination of inhibitors targeting distinct cell death pathways and assessed their impact on AMT-mediated tumor suppression. As depicted in Fig. S2A, B, combination of Z-VAD (an apoptosis inhibitors) or 3-Methyladenine (3-MA, an inhibitor of class III PI3K/Vps34) with AMT abrogated its inhibitory effect on glioma cell proliferation, while ferrostatin-1 (Fer-1, an effective and selective inhibitor of ferroptosis) exhibited no significant effect. To further assess whether the cytotoxic effect of AMT was associated with apoptosis, we first measured glioma cells by Annexin V/PI staining. Unsurprisingly, AMT greatly promoted apoptosis ratios in U87 and U251 cells (Fig. 2A). Additionally, as shown in Fig. 2B, immunoblotting was used to further analyze the proteins associated with apoptosis. After being treated with AMT, the expression of cleaved-PARP and cleaved-caspase 3 were significantly increased in the cancer cells tested. In brief, these results suggest that AMT induces apoptosis to inhibit glioma cell growth.

Many studies have proved that the important increase of intracellular ROS content can destruct the intracellular redox balance, which induces cell apoptosis in cancer cells [22, 23]. Then, we monitored whether AMT treatment increases ROS production via confocal laser scanning microscopy (CLSM) and flow cytometry with 2′,7′-dichlorofluorescein diacetate (DCFH-DA) as the fluorescence probe and observed a dramatically increased cellular ROS (Figs. 2C, D and S3A, B). Previous studies have demonstrated that ROS accumulation could further induce oxidative stress to cause mitochondrial damage [24]. The mitochondria are commonly served as key energy supplying organelles for tumor proliferation, and the specific damage to mitochondria leads directly to the decrease of mitochondrial membrane potential [25]. Fluorescence imaging results showed that JC-1 aggregates were extensively transformed into monomers with AMT treatment (Figs. 2E and S3C), which demonstrated that the mitochondrial membrane potential was decreased, leading to mitochondrial dysfunction. Apoptosis-related Bcl-2 family proteins have been reported to be associated with mitochondrial outer membrane integrity [26]. We subsequently evaluated the expression of related proteins. The results showed that the levels of Bcl-xL and Bcl-2 decreased in glioma cells after AMT treatment, while the expression of pro-apoptotic proteins Bax and Bak was promoted (Fig. 2F).

To further confirm ROS-induced apoptosis, U87 and U251 cells were treated with the ROS scavenger (NAC, N-acetylcysteine) combination with AMT. Pre-treatment of cells with NAC significantly attenuated AMT-induced cleavage of caspase 3 and PARP (Figs. 2G and S3D).The cytotoxicity phenotype was also rescued by NAC in the CCK-8 assay (Figs. 2H and S3E) and colony formation (Figs. 2I, J and S3F, G), which further verified the ROS-induced therapeutic effect of AMT in glioma cells. Moreover, treatment with NAC also decreased cytotoxicity which was supported by the LDH release assay (Figs. 2K and S3H). Collectively, these findings suggest that AMT inhibit the growth of glioma cells by inducing ROS-related apoptosis.

### AMT induces lethal autophagy arrest in glioma cell

**Fig. 3:**
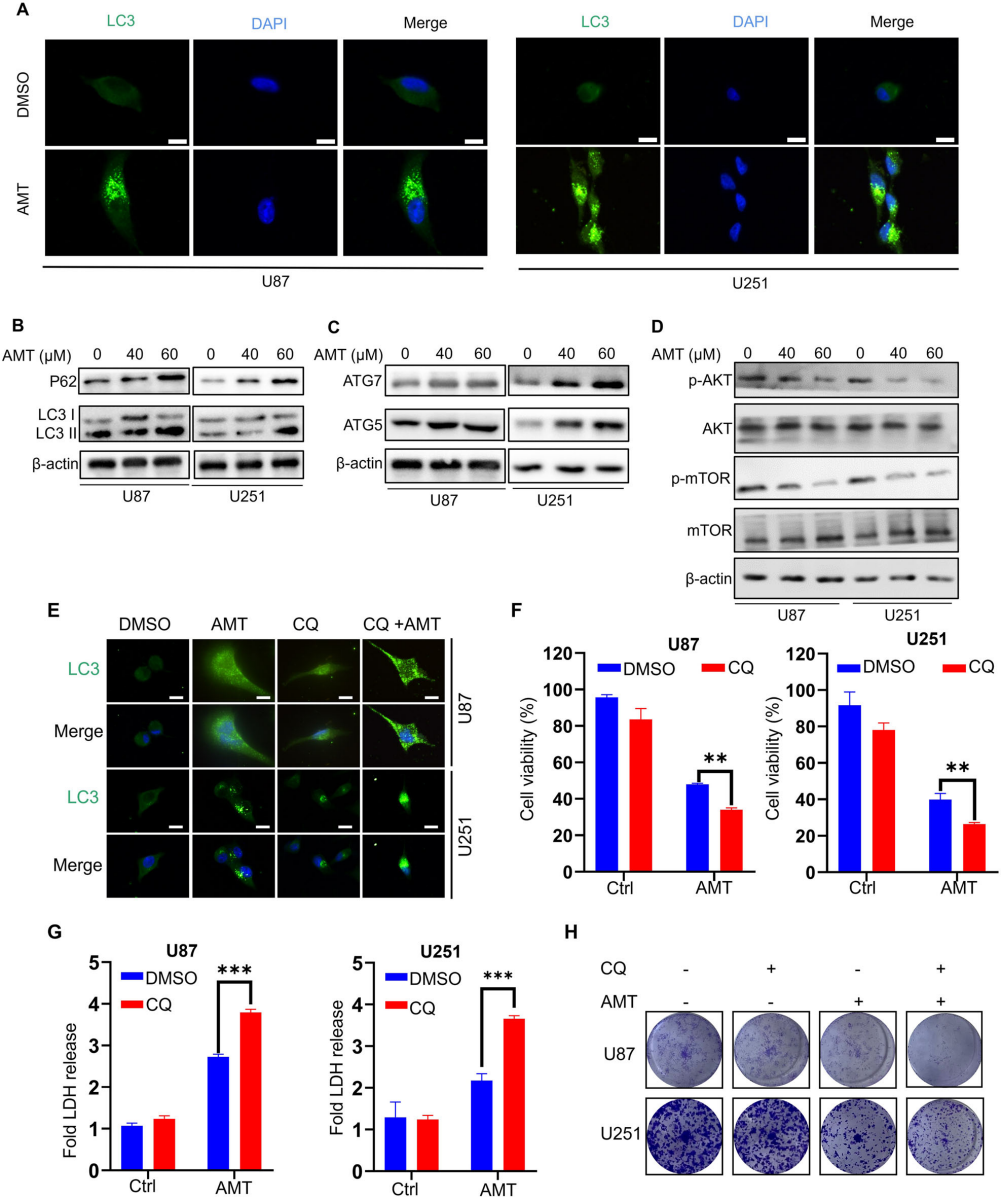
AMT induces autophagy in glioma cells. **A** Immunofluorescent analysis of endogenous LC3 puncta in cells treated with or without AMT for 24 h. Scale bar: 10 μm. **B**, **C** Immunoblotting analysis of P62 expression and LC3 turnover, ATG7 and ATG5 in U87 and U251 cells treated with AMT for 24 h. **D** Immunoblotting analysis of AKT, p-AKT, mTOR, and p-mTOR, cells were treated with AMT for 24 h. **E** U87 and U251 cells were treated with AMT (40 μM) alone or in combination with 5 μM CQ for 24 h, and LC3 spots were detected by immunofluorescence assay. Scale bar 50 μm. **F** Cell growth of U87 and U251 cells treated with AMT (40 μM) alone or in combination with 5 μM CQ for 24 h (*n* = 3). **G** LDH release from U87 and U251 cells treated as in (**E**) for 24 h (*n* = 3). **H** Colony formation of U87 and U251 cells treated as in (**E**) for 24 h. Error bars indicate mean ± SD (**P < 0.01, ***P < 0.001).

In addition, as previously mentioned that an autophagy inhibitor (3-MA) could also restore the inhibitory effect of AMT on glioma growth (Fig. S2A, B), suggesting that autophagy may be stimulated. We, therefore, wondered whether autophagy process is regulated by AMT in glioma cells. Firstly, we detected the expression of autophagy-related proteins after AMT treatment. According to the immunoblotting analysis, we found that the levels of autophagy-related proteins changed significantly after AMT treatment. One of the hallmarks of autophagy is the binding of LC3 I to PE and its conversion to LC3 II, which is required for autophagosome formation [27]. Autophagy was further confirmed by the increase of LC3 punctate structures in glioma cells after AMT treatment (Fig. 3A). Meanwhile, we observed that LC3 accumulation and P62 levels were increased in AMT-treated cells (Fig. 3B), suggesting the occurrence of autophagy and the blocking of autophagic flow. Furthermore, we found that AMT treatment enhanced the expression of ATG5 and ATG7 in a dose-dependent manner (Fig. 3C). As a classical signaling pathway, AKT/mTOR pathway has also been reported to mediate autophagy induced by antineoplastic drugs. Next, we investigated the effect of AMT-generated ROS on Akt/mTOR signaling pathway. Immunoblotting analysis showed that AMT reduced Akt and mTOR phosphorylation in a concentration-dependent manner (Fig. 3D). Moreover, in glioma cells, NAC also induced AMT-reduced P62 expression, decreased the LC3 II/LC3 I ratio, and reversed AMT-inhibited AKT/ mTOR phosphorylation (Fig. S4A). Taken together, these results indicate that AMT-induced ROS production blocks the Akt/mTOR signaling pathway.

Next, we sought to confirm the biological role of AMT in the regulation of autophagic flow in glioma cells. Moreover, 3-MA combined with AMT treatment significantly restored LC3 punctate aggregation after AMT treatment in glioma cells (Fig. S5A). Overall, these data indicate that AMT treatment initiates autophagy in glioma cells. Since the accumulation of LC3 II may be due to enhanced initiation of autophagy or impaired degradation of late autophagy [28], we further explored the potential mechanism by which AMT induces autophagosome accumulation in glioma cells. We examined changes in the punctate structure of endogenous LC3 in combination with chloroquine (CQ, an inhibitor of autophagosome-lysosome fusion). As shown, cells treated with CQ exhibited a stronger LC3 signal (autophagosome), implying increased autophagosomes and impaired autophagic flow in glioma cells (Fig. 3E). Taken together, these findings suggest that AMT inhibits the fusion of autophagosomes with lysosomes and induces an incomplete autophagic flow in glioma cells.

To investigate the impact of autophagy on the anti-glioma effect of AMT, we conducted experiments where glioma cells were exposed to a combination of AMT and either CQ or 3-MA. Our findings from CCK-8 (Fig. 3F), LDH (Fig. 3G), and clone formation assays (Fig. 3H) revealed that CQ disrupted the fusion between autophagosomes and lysosomes, leading to an accumulation of autophagosomes. This significantly worsened the growth inhibition induced by AMT. Conversely, when autophagy initiation and subsequent formation of autophagosomes were inhibited by 3-MA in AMT-treated glioma cells (Fig. S5B–D), it notably restored cell growth. In summary, our results indicate that AMT hinders glioma cell growth by promoting the accumulation of autophagosomes.

### AMT exhibits anti-tumor effect against glioma cancer in vivo

**Fig. 4:**
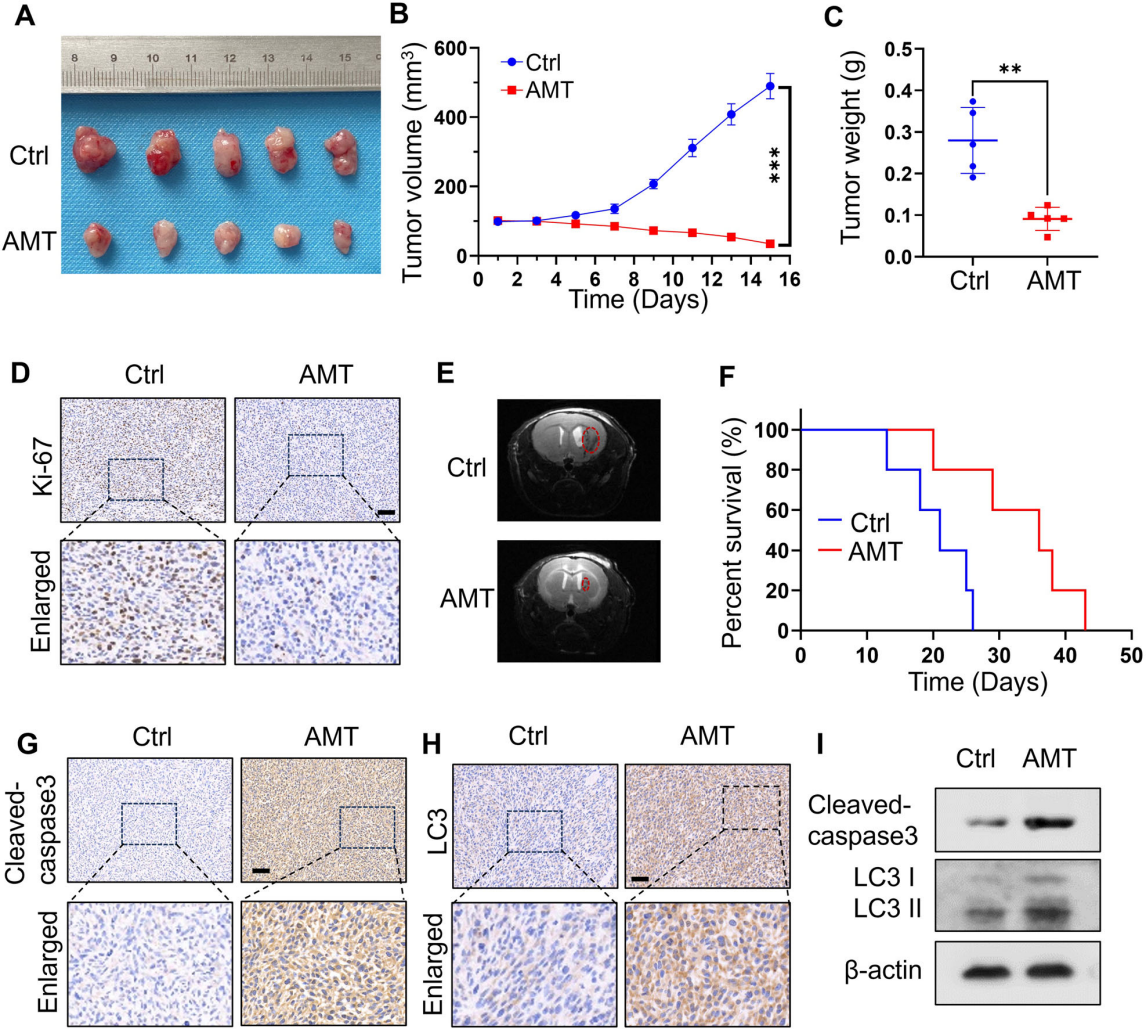
In vivo antitumor therapeutic efficacy of AMT. **A** Representative tumor photos of the U251 tumor-bearing mice with different treatments (*n* = 5). **B** The tumor volumes of mice at indicated time points (*n* = 5). **C** The tumor weight of mice at indicated time points (*n* = 5). **D** Immunohistochemical staining of Ki-67. Scale bar: 50 μm. **E** Representative MRI image of tumors in the glioma orthotopic mouse model. **F** Kaplan–Meier curves of glioma orthotopic mice from (**E**) (*n* = 5). **G**, **H** Immunohistochemical staining of cleaved-caspase3 and LC3 in tumor tissues after different treatments. Scale bar: 50 μm. **I** Immunoblotting analysis of the cleaved-caspase3 and LC3 levels in these tumor tissues. Error bars indicate mean ± SD (**P < 0.01, ***P < 0.001).

The in vitro antitumor growth of AMT encouraged us to investigate the in vivo antitumor efficacy of AMT, a mouse xenograft model was generated by subcutaneously inoculating the U251-bearing mice. The results of photos, tumor volumes, and tumor weight shown in Fig. 4A–C supported that the tumor growth process was significantly inhibited by AMT treatment compared with the control group. To further evaluated the anticancer ability of the AMT tumor tissues of each group were stained with the immunohistochemistry staining of Ki-67 (Fig. 4D). The destruction of tumor slices was realized in AMT group and there was almost no cancer cell proliferation compared with control group. In addition, we investigated the anti-glioma effect of AMT in an orthotopic xenograft mouse model. MRI analysis showed a significant reduction in glioma size on day 15 after treatment with AMT (Fig. 4E). Compared with the control group, the AMT group significantly prolonged the survival ratio of tumor-bearing mice (Fig. 4F). To further illuminate the antitumor mechanism of AMT in vivo, cleaved-caspase3 and LC3 expressions in tumors were examined through immunohistochemistry. As shown in Fig. 4G–H, consistent with the in vitro results, AMT-treated xenografts exhibited increased cleaved-caspase3 and LC3 expression. Besides, we also examined the expression levels of cleaved-caspase3 and LC3 in tumor tissues (Fig. 4I). These results showed that AMT could efficiently inhibit cancer cell growth via activating apoptosis/autophagy pathways.

### The biosafety of AMT in vivo

**Fig. 5:**
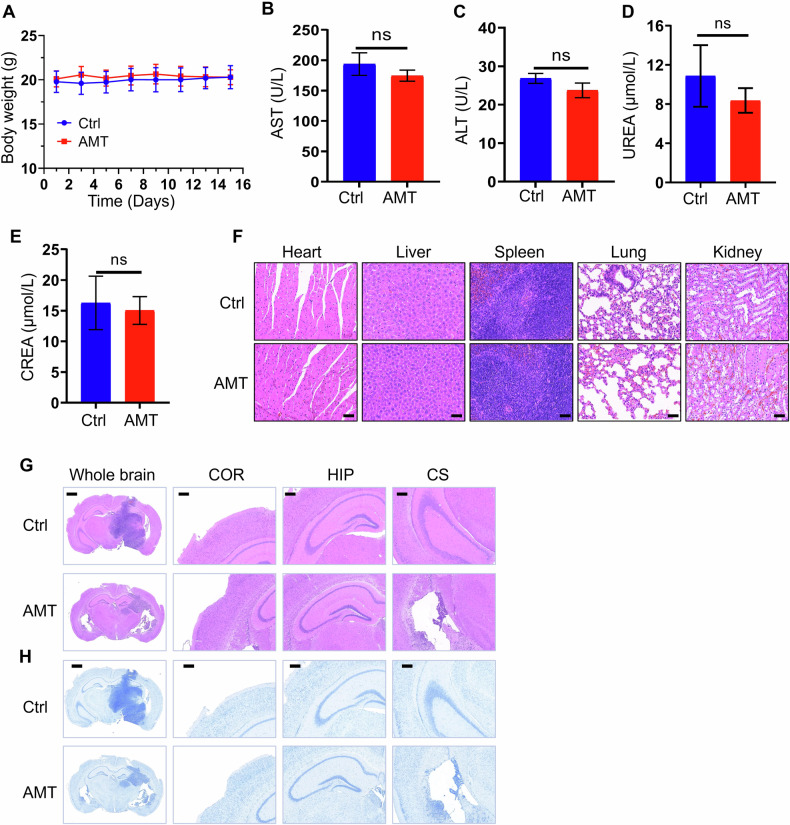
Evaluation of the biosafety of AMT in vivo. **A** Body weights of the U251 tumor-bearing mice after varied treatments (*n* = 5). Blood biochemical markers: AST (**B**); ALT (**C**); UREA (**D**); CREA (**E**) (*n* = 5). **F** H&E staining of the heart, liver, spleen, lung and kidney in mice treated with control or AMT. Scale bar: 50 μm. **G** Representative histological images of HE staining are representative of whole brain tissues (scale bar: 1 mm) and different regions (scale bar: 200 μm). **H** Representative histological images of the whole brain (scale bar: 1 mm) and different regions (scale bar: 200 μm) with Nissl staining. Cortex (COR), Hippocampus (HIP), Corpus Striatum (CS). Error bars indicate mean ± SD (no significant (ns), *p* > 0.05).

In addition, no significant body weight changes were observed in the treatment relative to the control group, suggesting the excellent biosafety profile of the AMT (Fig. 5A). Moreover, there was no evident change in the levels of varied serum biochemical parameters (Fig. 5B–E). Major organs (heart, liver, spleen, lung and kidney) through the H&E staining assay exhibited no obvious change of pathologic features after AMT administration (Fig. 5F). In addition, neurotoxicity is an important part of the non-clinical safety evaluation of drugs. It is well known that histopathological examination of nervous system tissues (such as cerebral cortex, hippocampus and striatum) is used to determine the specific site and extent of nerve damage. Neurons in the cortex (COR), hippocampus (HIP) and corpus striatum (CS) of mice treated with AMT exhibited orderly arrangement, with no apparent nuclear pyknosis observed (Fig. 5G). Furthermore, there was no significant alteration in the number of Nissl staining neurons in certain brain regions, and no reduction in Nissl bodies was detected (Fig. 5H). These results suggested that AMT at this dose could effectively inhibit tumor growth with good biosafety and low toxicity in vivo, and possess promising potential for glioma therapy.

## Discussion

Glioma is the most common primary intracranial malignant tumor, and the current clinical treatment methods mainly include surgery, chemoradiotherapy, novel molecular targeted therapy and immunotherapy. However, due to the resistance of glioma and the blood-brain barrier, these treatment methods have not brought significant efficacy to patients and the recurrence rate of glioma is very high [29–33]. Therefore, there is an urgent need to develop new therapeutic strategies to extend the survival time and improve the quality of life of glioma patients.

The research and development of a new drug faces many drawbacks such as huge human and material investment costs, a long research and development cycle, and uncertain adverse reactions of new drugs, so drug repurposing which refers to identifying new indications of approved drugs is becoming a hot topic. Therefore, drug repurposing can significantly reduce the cost, shorten research and development cycle and show many clear side effects [34]. At the same time, drug repurposing can also relieve the economic pressure of patients and solve the situation of drug availability faced by patients [35]. For example, aspirin, used for antipyretic, analgesic, anti-inflammatory and antiplatelet aggregation, has recently been found to restrain the growth of breast tumor cells and inhibit self-renewal of breast cancer stem cells [36]. Based on drug repurposing, amantadine, used in the treatment of influenza virus and Parkinson’s disease, has been applied to the therapeutic of melanoma and liver cancer in recent studies. In this study, we found that amantadine has good antitumor activity in glioma, and it may be used in clinical treatment. Although amantadine expresses the function of antitumor by mainly promoting apoptosis in melanoma and liver cancer, we found that amantadine can both trigger apoptosis and induce autophagy to inhibit the growth of glioma.

At present, the application of apoptosis in the development and treatment of tumor has been widely concerned, and numerous drugs perform an anti-tumor role by inducing cell apoptosis. For example, kaempferol inhibits the proliferation of glioma cells by promoting apoptosis [37]. This study found that there was significant apoptosis of glioma cells after amantadine administration, which may be one of the reasons why amantadine inhibits glioma growth. The processes involved in apoptosis are quite complex, which involve exogenous and endogenous pathways, and the destruction of mitochondria is an important link to trigger endogenous pathways. In the present study, we found that amantadine caused a large accumulation of intracellular ROS and changes of mitochondrial membrane potential in glioma cells. At the same time, the killing effect of amantadine on glioma cells could be significantly reversed by ROS inhibitor NAC. Therefore, amantadine may induce oxidative stress by promoting intracellular ROS accumulation, and then trigger endogenous apoptosis by destroying mitochondrial structure, thereby inhibiting the growth of glioma cells. In addition, when amantadine generates intracellular oxidative stress, it may lead to DNA damage, and then disrupt the expression of signal molecules in apoptosis-related signaling pathways to induce apoptosis in glioma cells.

Autophagy, as a hotspot of contemporary research, has different effects in various tumors and numerous chemotherapy drugs. For example, paclitaxel or 5-FU induces protective autophagy in tumor cells, so autophagy inhibitors enhance the anti-tumor effect of drugs [38, 39] In contrast, several drugs kill tumor cells mainly by inducing cytotoxic autophagy, thus autophagy inhibitors reverse the anti-tumor activity of drugs [40]. In this study, we found that amantadine promoted the initiation of autophagy and blocked the fusion of autophagosome and lysosome into autolysosome, which accumulated a large number of autophagosomes in the cells and then suppressed the development of glioma cells. The autophagy initiation inhibitor 3-MA reversed the antitumor effect of amantadine, while the late autophagy inhibitor CQ enhanced the killing effect of amantadine. This suggested that autophagosome accumulation is the key to amantadine in the process of anti-glioma. Therefore, promoting the initiation of autophagy and inhibiting the formation of autolysosomes are anticipated to be a new direction of tumor therapy.

## Conclusion

**Fig. 6:**
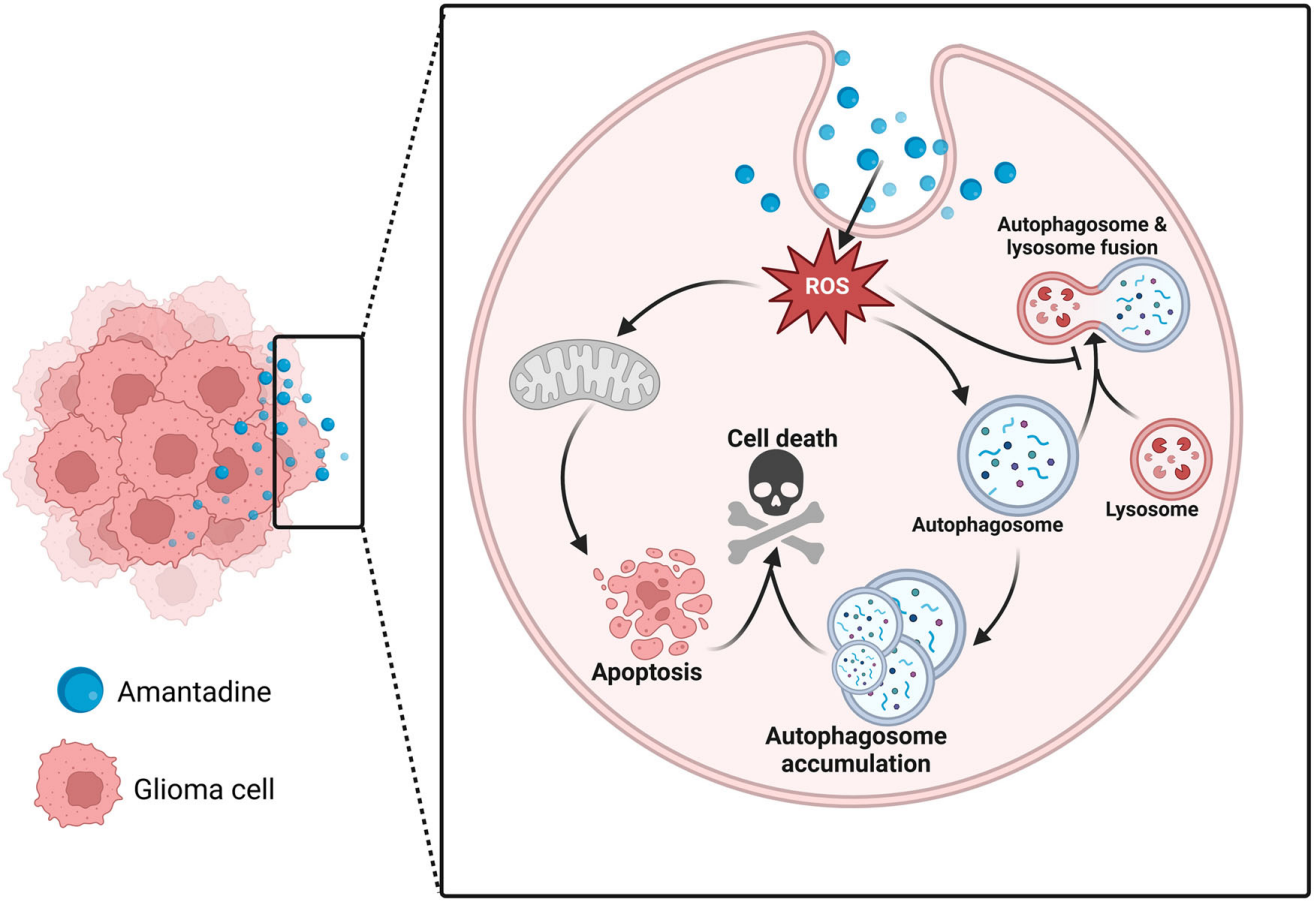
Schematic model for the mechanism of AMT. AMT promotes the excessive ROS accumulation and subsequent apoptosis in glioma cells. Moreover, AMT induces the accumulation of autophagosomes, thereby inducing cell-killing autophagy.

In summary, we can propose a model in which amantadine might be an effective antitumor agent by regulating apoptosis and autophagy in glioma cells (Fig. 6). It is worth noting that amantadine induces mitochondrial destruction by promoting ROS accumulation, thus triggering glioma cell apoptosis. At the same time, amantadine plays an anti-glioma role by inducing initiation of autophagy and inhibiting the fusion of autophagosome and lysosome. These findings reveal that amantadine inhibits glioma by inducing apoptosis and autophagy, which establishes a promising strategy for the treatment of glioma by exploring novel indications of amantadine.

## Supplementary information

Supplemental figures

Uncropped original western blots

## Data Availability

All data generated or analyzed during this study are included in this published article.
