# Supplementary material for: Amantadine against glioma via ROS-mediated apoptosis and autophagy arrest
Source: Cell Death Dis. 2024 Nov 15;15(11):834. doi: 10.1038/s41419-024-07228-x (PMC11568115; doi:10.1038/s41419-024-07228-x)
Supplement: Supplementary file 1 — Supplemental figures [file 41419_2024_7228_MOESM1_ESM.docx]

**Supplemental Figures**

**Amantadine against glioma via ROS-mediated apoptosis and autophagy arrest**

Yusong Luo^1, 2†^, Ruolan Liu^1, 2†^, He Zhang^1, 2^ Hongyu Wang^1, 2^, Hang Yin^1, 2^, Guopeng Tian^1, 2^, Bo Wang^1, 2^, Yunji Yan^1, 2^, Zilin Ding^1, 2^, Junqiang Dai^1, 2^, Liang Niu^1, 2^, Guoqiang Yuan^1, 2*^ and Yawen Pan^1, 2, 3*^

Affiliations:

^1^ Department of Neurosurgery, the Second Hospital & Clinical Medical School, Lanzhou University, Lanzhou 730030, China*.*

^2^ Gansu Provincial Clinical Research Center for Neurological Diseases, the Second Hospital & Clinical Medical School, Lanzhou University, Lanzhou 730030, China*.*

^3^ Academician Workstation, the Second Hospital & Clinical Medical School, Lanzhou University, Lanzhou 730030, China.

† These authors contributed equally to this work.

* Correspondence: yuangq08@lzu.edu.cn; [yawen_pan@126.com](mailto:yawen_pan@126.com)

**Supplemental figures**


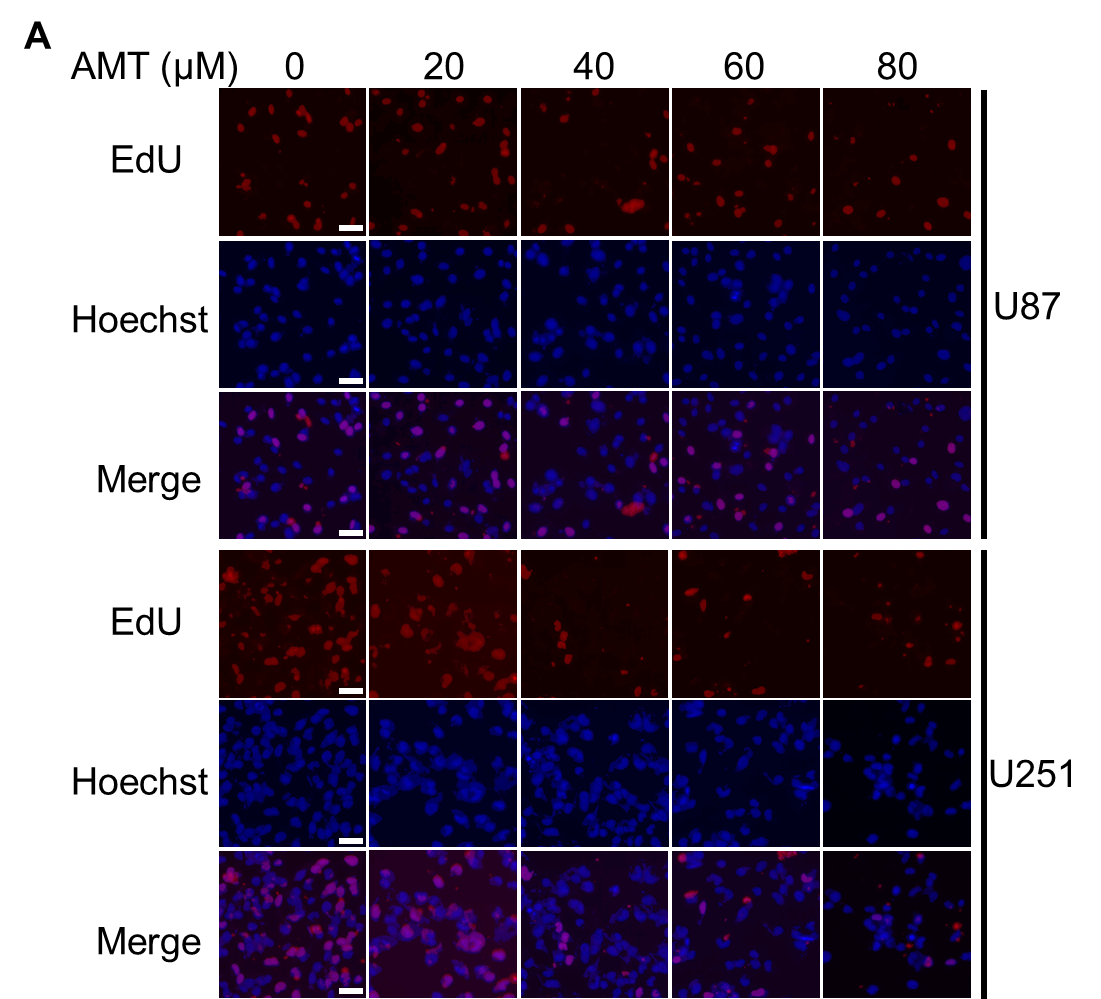


**Figure S1.** **AMT repressed the cell viability of glioma cells.** (**A**) Representative images of the EdU assay of U87 and U251 cells treated with indicated concentrations of AMT for 24 h. Scale bar:50 μm.


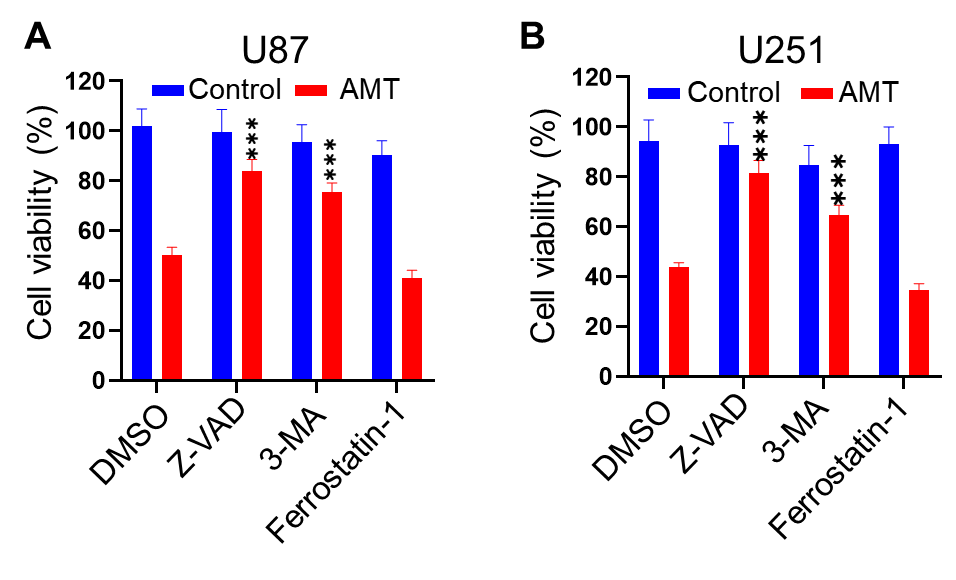


**Figure S2. AMT suppresses glioma cells dependently of apoptosis and autophagy.** (**A-B**) CCK-8 assay of U87 (A) and U251(B) cells treated with or without AMT (40μM), in the presence or absence of three different patterns inhibitors of cell death (Z-VAD, 4 μM; 3-MA, 5 mM; Ferrostatin-1, 20 μM) for 24 h (n=3). Error bars indicate mean ± SD (***P<0.001).


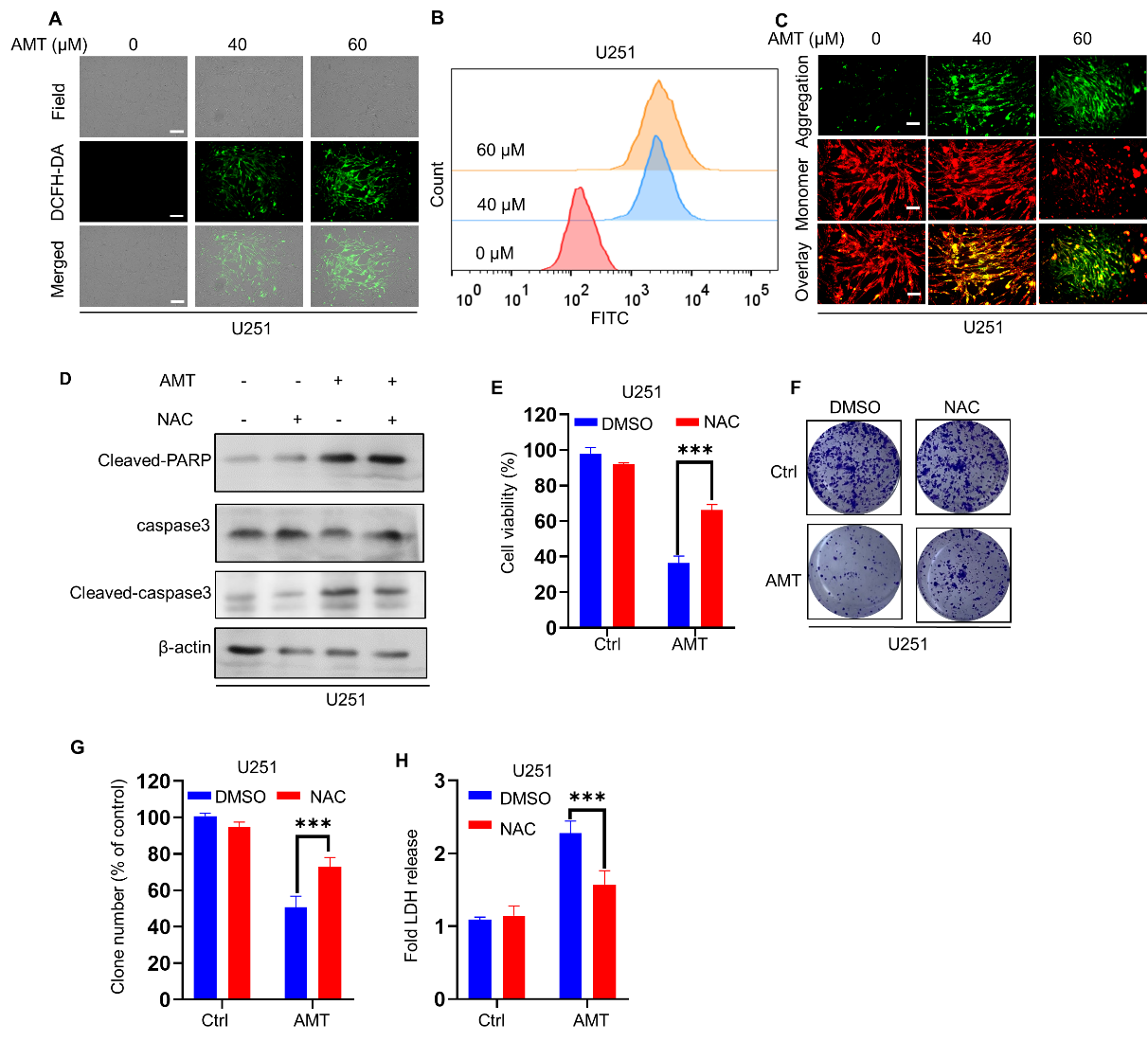


**Figure S3. AMT-induced ROS accumulation contributes to its anti-glioma activity.** (**A-B**) ROS generation in U251 cells after different treatments using fluorescence imaging (A) and flow cytometry (B). Scale bar: 100 μm. (**C**) JC-1 imaging in U251 cells after different treatment. Scale bar: 100 μm. (**D**) Immunoblotting analysis of cleaved-PARP, caspase3 and cleaved caspase3 in U251, cells were treated with 40 μM AMT in the presence or absence of NAC (2 mM). (**E**) Cell viability of U251 cells treated with 40 μM AMT in the presence or absence of NAC (2 mM) (n=3). (**F-G**) Colony formation assay of U251 cells (n=3). (**H**) LDH release assay of U251, cells were treated as in (D) for 24 h (n=3). Error bars indicate mean ± SD (***P < 0.001).

**
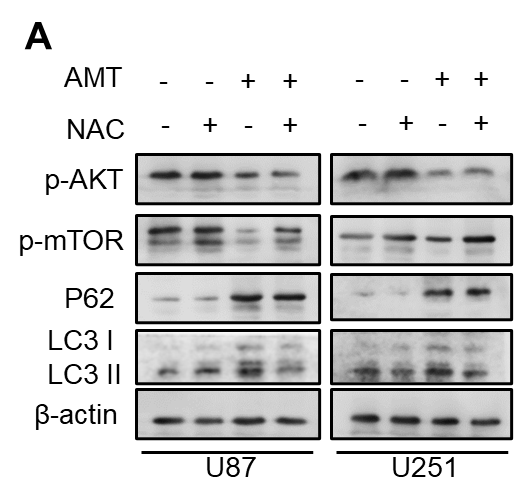
**

**Figure S4.** **AMT induces autophagy via /Akt/mTOR signaling** **by triggering ROS production. (A)** Immunoblotting analysis of p-AKT, p-mTOR, P62 and LC3, cells were treated with 40 μM AMT in the presence or absence of NAC (2 mM).

**
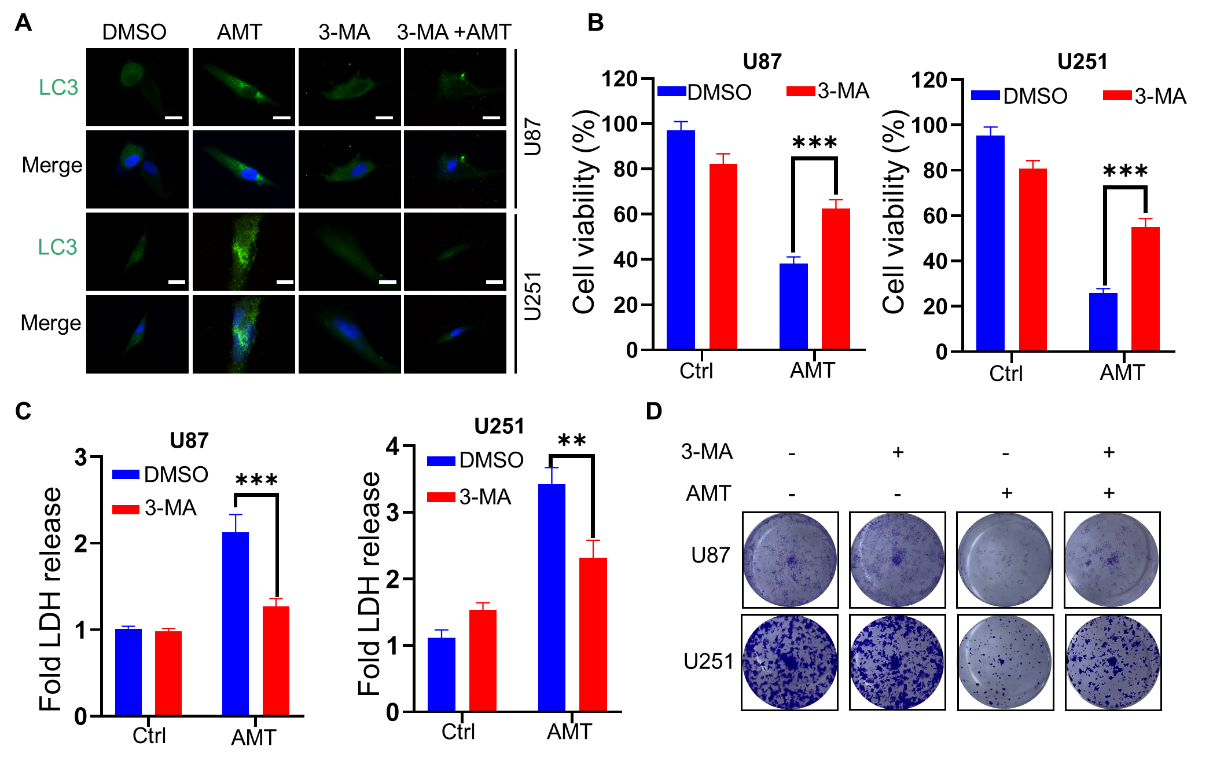
**

**Figure S5. AMT induces autophagy initiation.** (**A**) U87 and U251 cells were treated with AMT (40 μM) alone or in combination with 5mM 3-MA for 24 h, and LC3 spots were detected by immunofluorescence assay. Scale bar 50µm. (**B**) Cell growth of U87 and U251 cells treated with AMT (40 μM) alone or in combination with 3-MA for 24 h (n=3). (**C**) LDH release from U87 and U251 cells treated with AMT (40 μM) or in combination with 5mM 3-MA for 24 h (n=3). (**D**) Colony formation of U87 and U251 cells treated with AMT (40 μM) alone or in combination with 3-MA. Error bars indicate mean ± SD (**P < 0.01, ***P < 0.001).
