# Supplementary material for: Amantadine against glioma via ROS-mediated apoptosis and autophagy arrest
Source: Cell Death Dis. 2024 Nov 15;15(11):834. doi: 10.1038/s41419-024-07228-x (PMC11568115; doi:10.1038/s41419-024-07228-x)

Fig. 2B

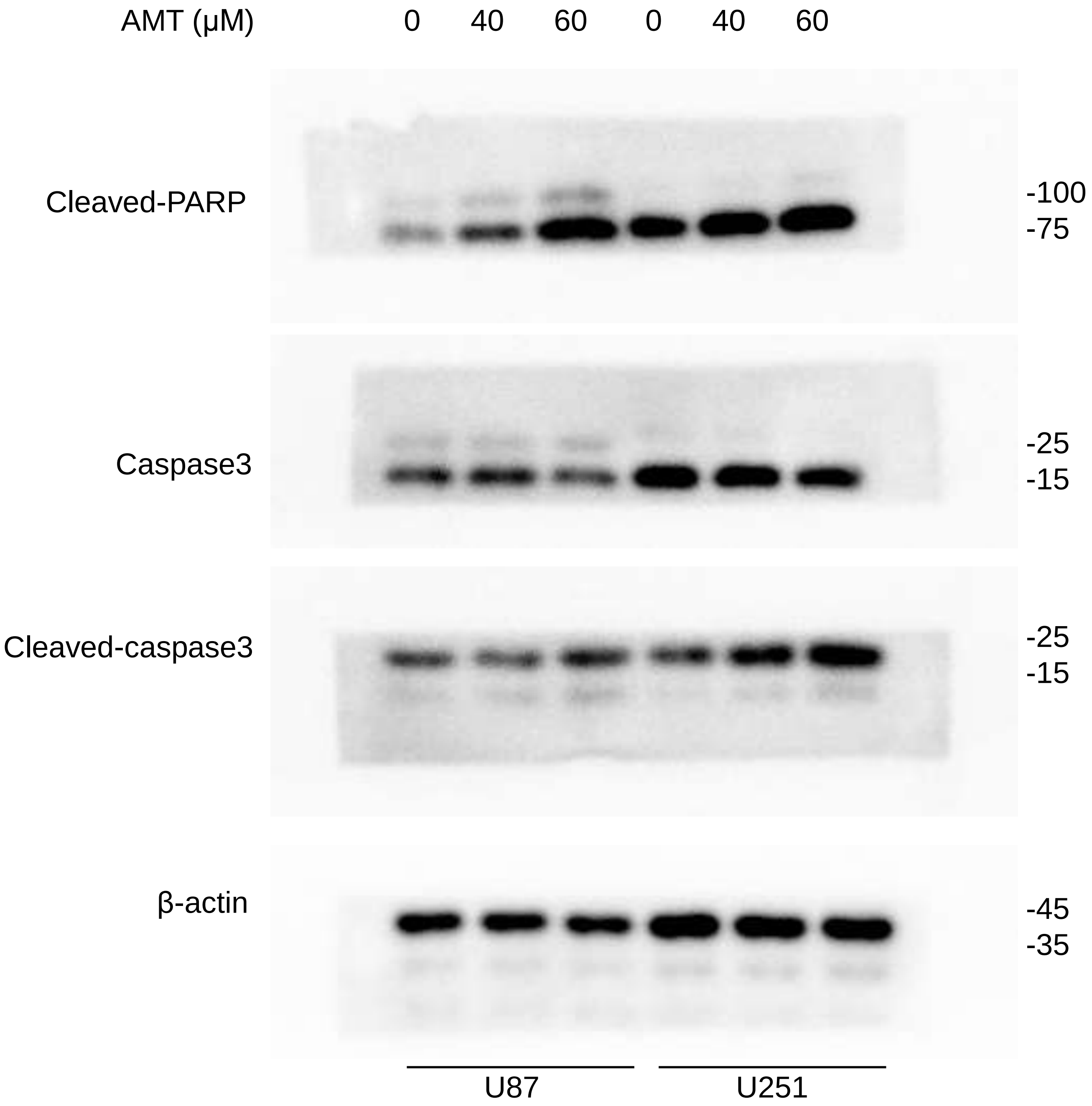

AMT (μM)
0
40
60
0
40
60
Cleaved-PARP
-100
-75
Caspase3
-25
-15
Cleaved-caspase3
-25
-15
β-actin
-45
-35
U87
U251

Fig. 2F

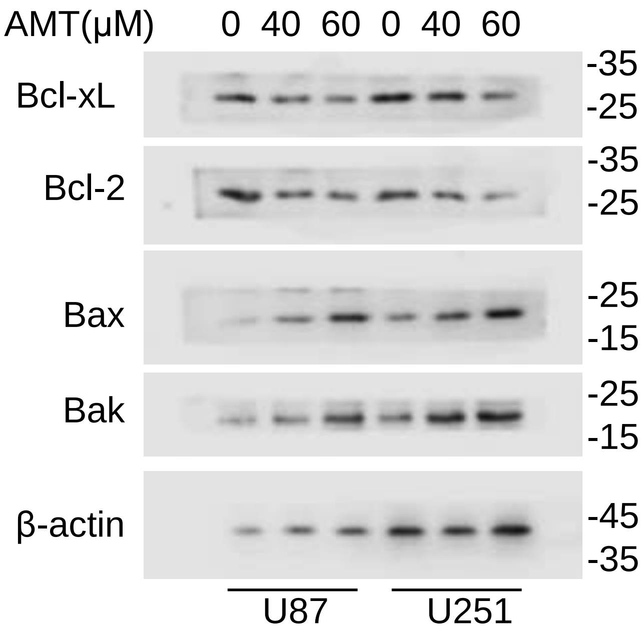

AMT(μM) 0 40 60 0 40 60
Bcl-xL
Bcl-2
Bax
Bak
β-actin
-35
-25
-35
-25
-25
-15
-25
-15
-45
-35
U87
U251

Fig. 2G

Fig.S3D

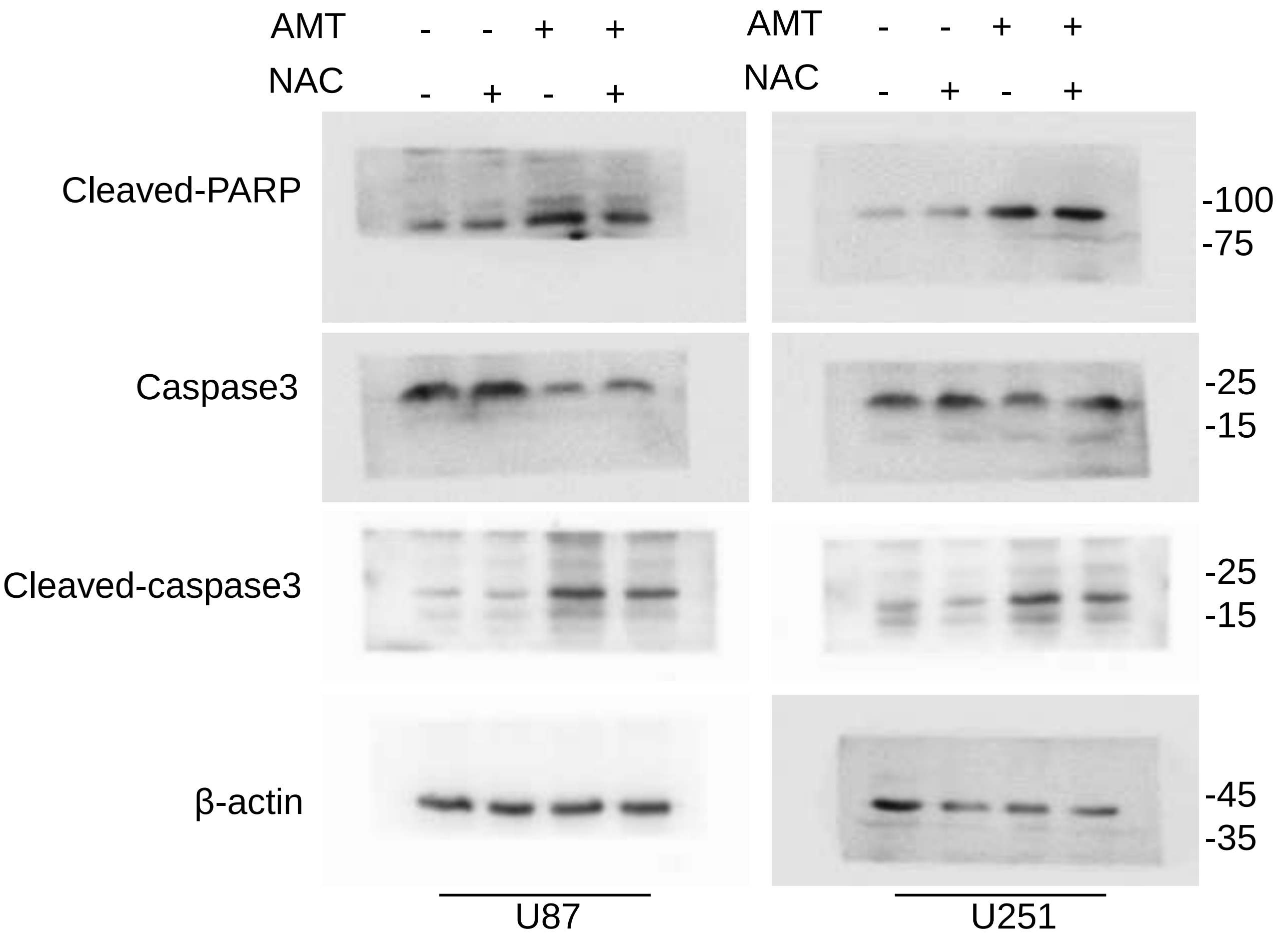

Fig. 3B

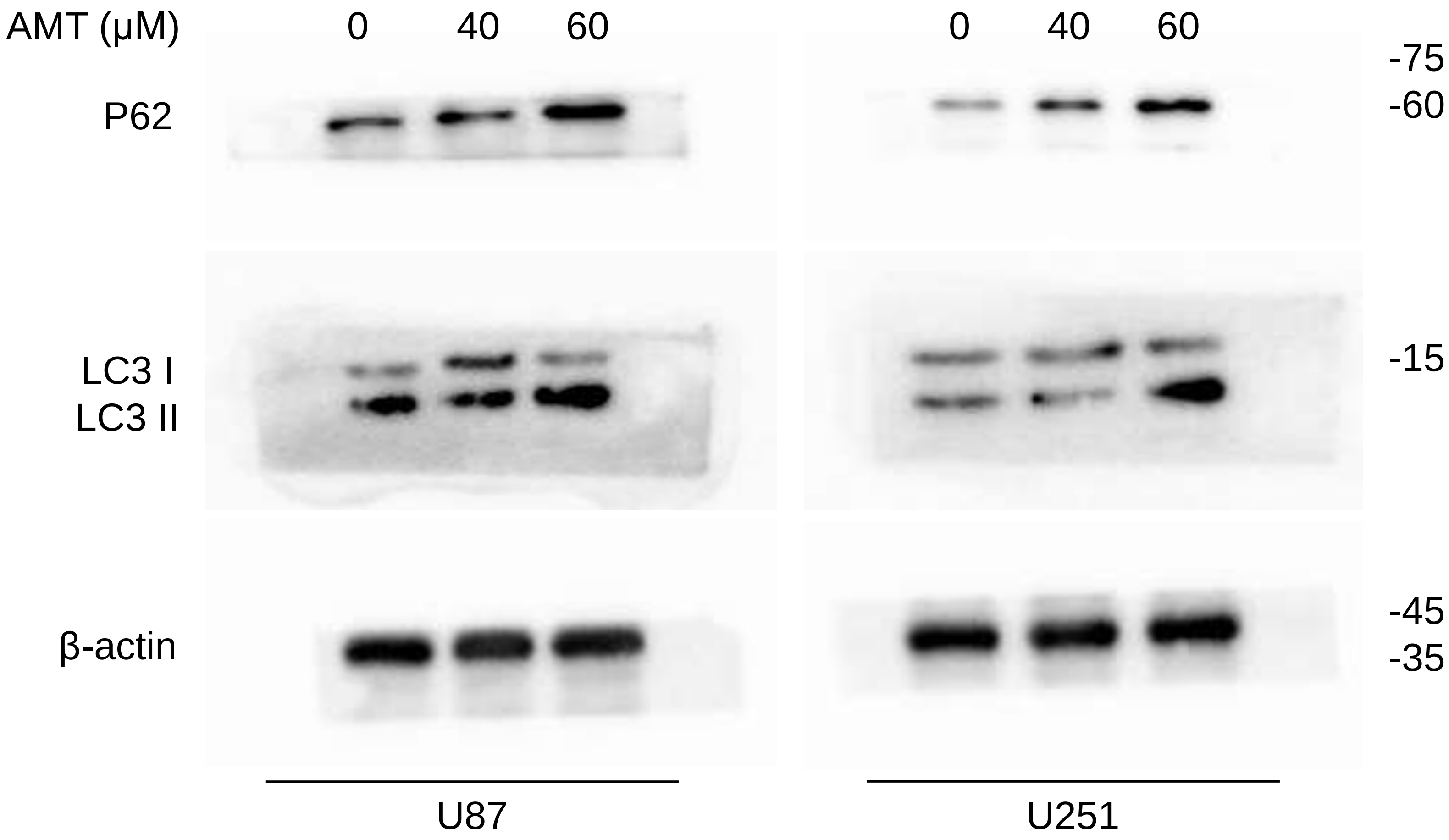
AMT (μM)
0
40
60
0
40
60
-75
P62
-60
LC3 I
LC3 II
-15
β-actin
-45
-35
U87
U251

Fig. 3C

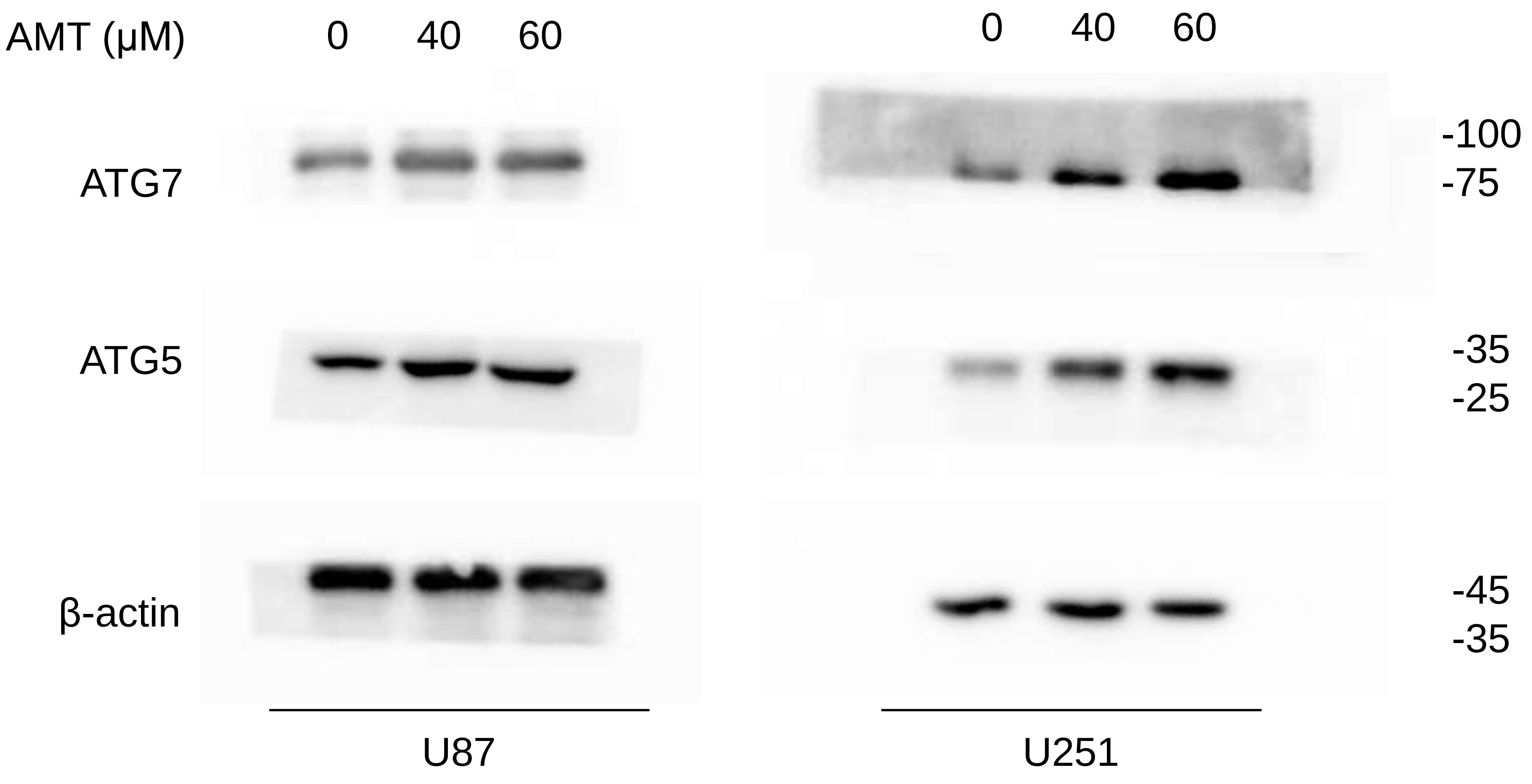

Fig. 3D

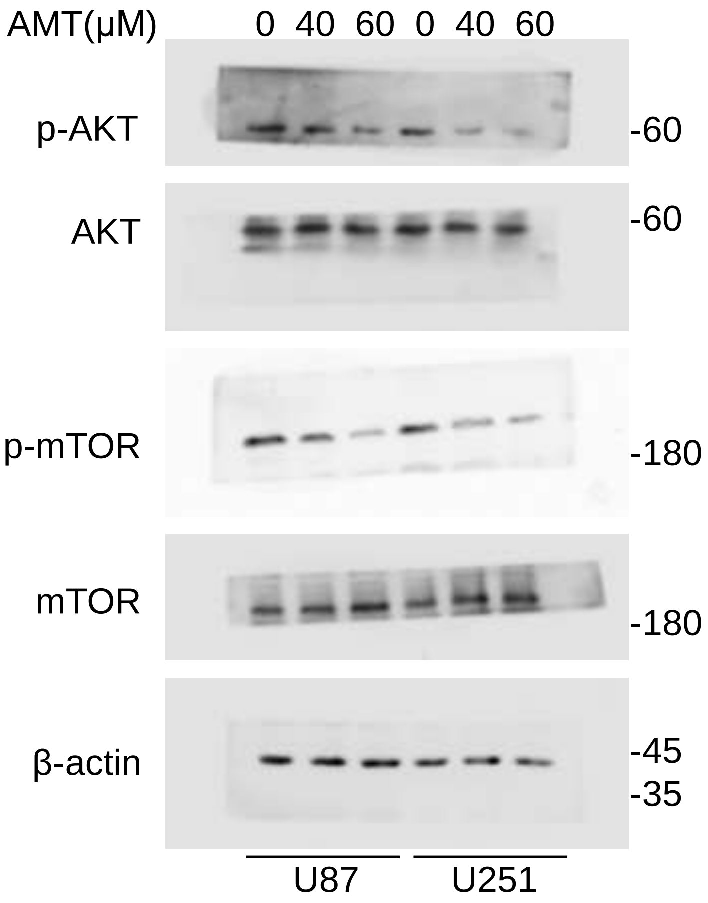

AMT(μM)
0 40 60 0 40 60
p-AKT
-60
AKT
-60
p-mTOR
-180
mTOR
-180
β-actin
-45
-35
U87
U251

Fig. S4A

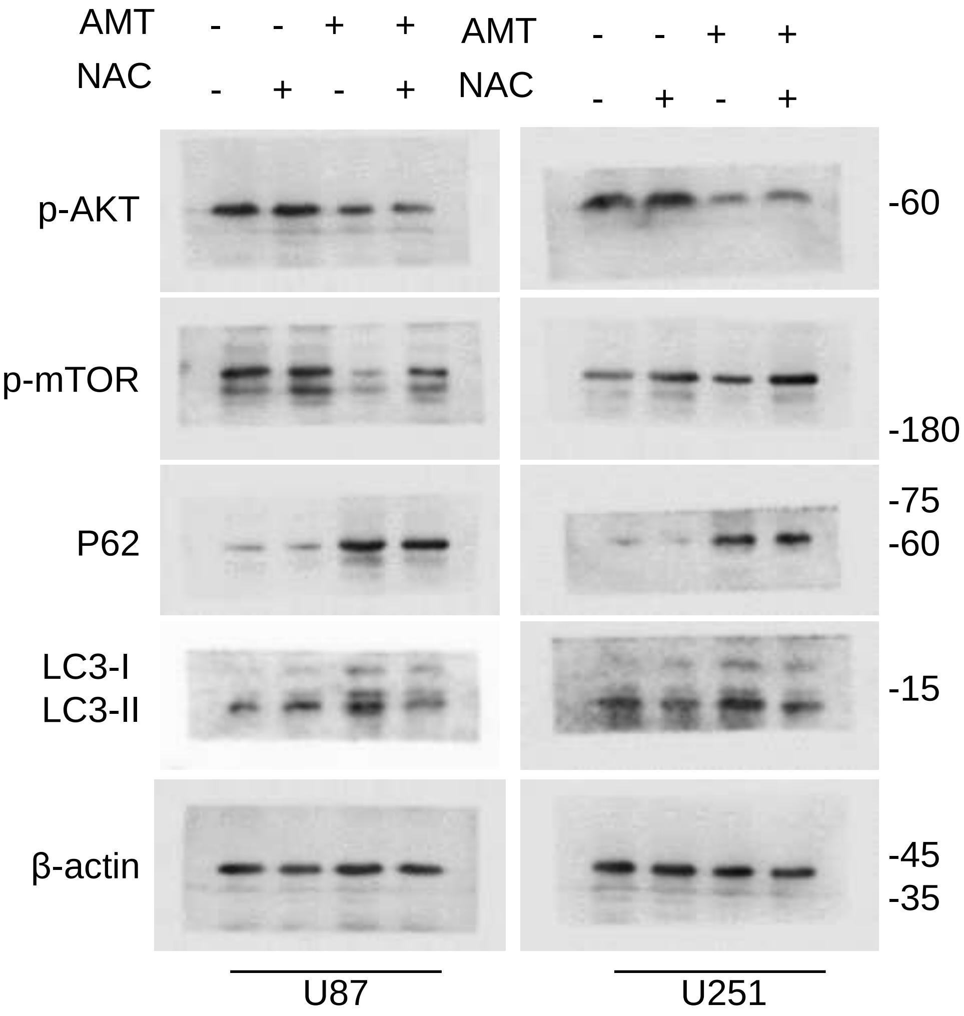

AMT - - + +
NAC - + - +
AMT - - + +
NAC - + - +
p-AKT
p-mTOR
P62
LC3-I
LC3-II
β-actin
-60
-180
-75
-60
-15
-45
-35
U87
U251

Fig. 4I

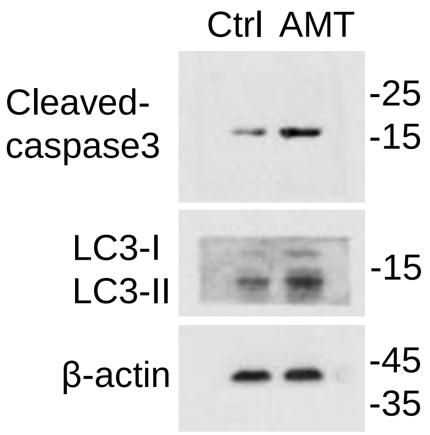

Supplement: Supplementary file 2 — Uncropped original western blots [file 41419_2024_7228_MOESM2_ESM.pdf]
